# The Viscoelastic Swirled Flow in the Confusor

**DOI:** 10.3390/polym13040630

**Published:** 2021-02-20

**Authors:** Aidar Kadyirov, Rinat Zaripov, Julia Karaeva, Ekaterina Vachagina

**Affiliations:** Institute of Power Engineering and Advanced Technologies, FRC Kazan Scientific Center, Russian Academy of Sciences, 420111 Kazan, Russia; rinat_zaripov.imm@mail.ru (R.Z.); julieenergy@list.ru (J.K.); vachaginae@mail.ru (E.V.)

**Keywords:** viscoelastic flow, Giesekus model, swirl, confusor, contraction, normal stress difference

## Abstract

A two-dimensional mathematical model for a steady viscoelastic laminar flow in a confusor was developed under the condition of swirled flow imposed at the inlet. Low density polyethylene was considered as a working fluid. Its behavior was described by a two-mode Giesekus model. The proposed mathematical model was tested by comparing it with some special cases presented in the literature. Additionally, we propose a system of equations to find the nonlinear parameters of the multimode Giesekus model (mobility factor) based on experimental measurement. The obtained numerical results showed that in a confusor with the contraction rate of 4:1, an increase in the swirl intensity at Wi < 5.1 affects only the circumferential velocity, while the axial and radial velocities remain constant. The distribution pattern of the first normal stress difference in the confusor is qualitatively similar to the one in a channel with abrupt contraction, i.e., as the viscoelastic fluid flows in the confusor, the value of N1 increases and reaches a maximum at the end of the confusor. Dimensionless damping coefficients of swirl are used to estimate the swirl intensity. The results show that the swirl intensity decreases exponentially.

## 1. Introduction

Investigation of viscoelastic fluid flows in channels of various configurations is of particular practical interest for polymer production. During extrusion, the polymer material passes through a channel with a screw, thus creating a swirling flow directed to the die nozzle. The nozzle geometry varies depending on the shape of the finished product, but a convergent channel (confusor) is an integral part of die [1]. Since we only consider the flow of initially swirled viscoelastic fluid in the convergent channel, our review is limited to the results covering this specific subject.

The literature mainly considers individual extreme cases, namely viscoelastic flows in channels with abrupt contraction (planar and axial symmetry flow), flow patterns in viscoelastic fluids flowing in a limited space with a rotating wall (bottom or upper lid, pipe surface), and cone-and-plate flows [2].

Interestingly, the research of viscoelastic fluid flows in channels with abrupt contraction is mainly concerned with planar flows, while the planar configuration is better suited to visualization studies through birefringence strand techniques and particle image velocimetry (PIV) [3,4]. Flow in a channel with abrupt contraction (as well as flow past a cylinder [5]) is a well-known benchmark problem used for the testing of reliability of new or modified numerical methods simulating the flows of viscoelastic fluid that employ multimodal rheological equations of state, e.g., the Giesekus model [6], the Phan-Thien–Tanner model (PTT) [7], Extended Pom-Pom [8], Oldroyd-B [9], and Fene-P [10]. A special feature of viscoelastic flows in channels with abrupt contraction is a recirculation zone, the size of which depends on the Weissenberg/Deborah number [11] and on extensional-flow properties [12]. The comprehensive review presented in [13] summarizes key factors influencing secondary entry flows for polymer melts, while outstanding issues in numerical methods and novel and challenging applications of viscoelastic fluids are discussed in [14].

Here, we consider a viscoelastic flow in a channel with a 4:1 contraction ratio because it has been extensively studied in the literature [12,15] and because it has been defined as benchmark geometry for the workshop on the numerical simulation of viscoelastic flow. For example, planar flow was investigated in [16,17,18,19,20,21,22,23,24,25], while axisymmetric flow was considered in [12,26,27,28,29,30].

Earlier [19], it was found that the planar contraction flow gives rise to very low vortex activity in the salient corner, unlike similar flows in circular contractions. Therefore, a circular channel is the most interesting geometry, particularly as far as swirled flows are concerned, because both the geometry and the system of equations describing hydromechanical processes are invariant to a circumferential angle *phi* (angle of rotation about a symmetry axis in a cylindrical coordinate system).

According to the literature, viscoelastic fluid flows in channels with abrupt contraction have been studied extensively for the case when a developed velocity profile (Newtonian or non-Newtonian) is set at the channel inlet, and when the inlet and outlet parts of the channel are long enough so as not to affect the flow pattern near the contraction.

Lately, embedded software (e.g., ANSYS-Polymat) has become an increasingly popular tool for the estimation of both the discrete spectrum of relaxation and non-linear parameters of differential PTT and Giesekus models [1,29]. According to preliminary analysis, our proprietary software approximates the viscosity curve with fewer modes and the same error of approximation of numerical and experimental data. Normal stresses exist in viscoelastic media, as discovered in experiments by Garner and Nissan [30] and interpreted by Weissenberg [31]. These results triggered the research of the structure of swirled viscoelastic flows in a confined cylinder with a rotating bottom lid [32,33,34,35,36,37]) or pipe wall [38]. Laminar pipe flow with a controllable wall swirl has been studied in [39] to explore the behavior of inelastic shear-dependent fluids. A vortex shedding regime was illustrated using experimental data in [35]. It was also observed that the dimensionless circumferential velocity decreases with the increase in the Weissenberg number, We [34]. The obtained results were employed for the development of advanced rotary rheometers with plate-plate and cone-plate measurement systems.

Three-dimensional numerical simulation [33], contrary to earlier experiments in [32], demonstrated that the structure of swirled flow in a confined cylinder with a rotating bottom lid is axisymmetric. It should be mentioned that numerical results obtained in [33] were validated by checking the stability criterion [40] for the case of highly unsteady spiral vortex flow of viscoelastic fluid. Thus, our two-dimensional approach to the construction of a mathematical model for a swirled flow of two-mode Giesekus fluid is consistent with the physical pattern of flow.

Numerical analysis [1] of screw swirling effects on fiber orientation in large area additive manufacturing polymer composite deposition is the most similar in its content to our study. The authors [1] considered a 3D problem using the exponential form of the Phan-Thien–Tanner model (PTT) and commercial software ANSYS PolyFlow. They had to use three-dimensional formulation because it is impossible to modify the standard rheological equation of state in ANSYS PolyFlow when using an axisymmetric problem statement to take fluid rotation into account.

The present work aims to develop a two-dimensional mathematical model of swirled viscoelastic fluid flow in a circular convergent channel (confusor). Using this model, the distribution of hydrodynamic parameters is obtained more easily, and it complies with the results obtained for a 3D problem.

## 2. Materials and Methods

**Figure 1:**
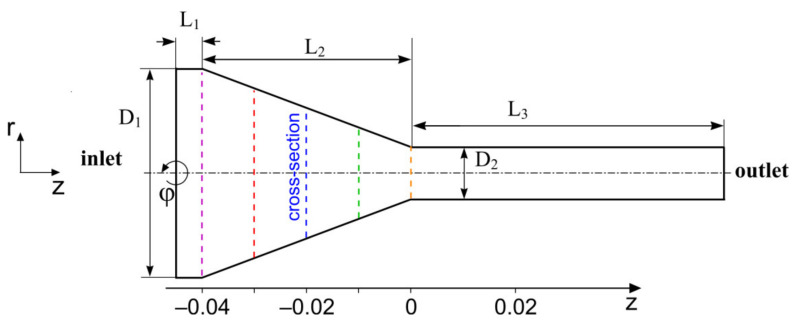
Sketch of the confusor geometry (contraction rate 4:1).

Let us consider a steady-state swirled flow of a two-mode viscoelastic fluid in a confusor with a contraction rate of 4:1 (Figure 1). At the inlet, the swirled flow is prescribed by the boundary conditions, which are invariant with respect to variable φ, so the main problem can be reduced to a two-dimensional form since the governing equations for the considered problem are also invariant to variable φ. Thereby, the mathematical model of viscoelastic fluid flow in the confusor is as follows:

(1) $$\rho_{f}\left(V_{r}\frac{\partial V_{r}}{\partial r}+V_{z}\frac{\partial V_{r}}{\partial z}-\frac{V_{\varphi }{}^{2}}{r}\right)=-\frac{\partial p}{\partial r}+\frac{1}{r}\frac{\partial }{\partial r}\eta_{s}\left(2r\frac{\partial V_{r}}{\partial r}\right)+\frac{\partial }{\partial z}\eta_{s}\left(\frac{\partial V_{r}}{\partial z}+\frac{\partial V_{z}}{\partial r}\right)-2\eta_{s}\frac{V_{r}}{r^{2}}\;+\sum_{m=1}^{n}\left(\frac{\partial \sigma_{rr(m)}}{\partial r}+\frac{\sigma_{rr(m)}}{r}+\frac{\partial \sigma_{rz(m)}}{\partial z}-\frac{\sigma_{\varphi \varphi (m)}}{r}\right),$$

(2) $$\rho_{f}\left(V_{r}\frac{\partial V_{\varphi }}{\partial r}+V_{z}\frac{\partial V_{\varphi }}{\partial z}+\frac{V_{r}V_{\varphi }}{r}\right)=\frac{1}{r^{2}}\frac{\partial }{\partial r}\eta_{s}\left(r^{2}\frac{\partial V_{\varphi }}{\partial r}-rV_{\varphi }\right)+\frac{\partial }{\partial z}\eta_{s}\frac{\partial V_{\varphi }}{\partial z}\;+\sum_{m=1}^{n}\left(\frac{\partial \sigma_{r\varphi (m)}}{\partial r}+2\frac{\sigma_{r\varphi (m)}}{r}+\frac{\partial \sigma_{\varphi z(m)}}{\partial z}\right),$$

(3) $$\rho_{f}\left(V_{r}\frac{\partial V_{z}}{\partial r}+V_{z}\frac{\partial V_{z}}{\partial z}\right)=\frac{\partial p}{\partial z}+\frac{1}{r}\frac{\partial }{\partial r}r\eta_{s}\left(\frac{\partial V_{r}}{\partial z}+\frac{\partial V_{z}}{\partial r}\right)+\frac{\partial }{\partial z}2\eta_{s}\frac{\partial V_{z}}{\partial z}\;+\sum_{m=1}^{n}\left(\frac{\partial \sigma_{rz(m)}}{\partial r}+\frac{\sigma_{rz(m)}}{r}+\frac{\partial \sigma_{zz(m)}}{\partial z}\right),$$

(4) $$\frac{\partial V_{r}}{\partial r}+\frac{V_{r}}{r}+\frac{\partial V_{z}}{\partial z}=0$$

where $V_{r}$, $V_{\varphi }$, $V_{z}$ are radial, circumferential, and axial velocity, respectively; r,φ,z are cylindrical coordinate system variables (axis z is the axis of rotation); *p* is pressure, $\rho_{f}$ is fluid density; $\sigma_{rr(m)}$, $\sigma_{r\varphi (m)}$, $\sigma_{rz(m)}$, $\sigma_{\varphi \varphi (m)}$, $\sigma_{\varphi z(m)}$, $\sigma_{zz(m)}$ are components of extra stress tensor (T); $T=\sigma_{ij}=\sum_{m=1}^{2}\sigma_{m}+\sigma_{N}$ is a total mode number that is equal to two, $\;\sigma_{N}=2\eta_{N}D$ is a Newtonian component of tensor T; $\eta_{N}$ is the viscosity of $\sigma_{N}$.

Boundary conditions:

(5) $$V_{r}=0,\;V_{\varphi }=\frac{K\cdot V_{a}}{R_{1}}\cdot r,\;V_{z}=\frac{2V_{a}}{R_{1}^{2}}\left(R_{1}^{2}-r^{2}\right)\;(\mathrm{at}\;\mathrm{the}\;\mathrm{inlet})$$

(6) $$V_{r}=0,\;V_{\varphi }=0,\;V_{z}=0\;(\mathrm{at}\;\mathrm{the}\;\mathrm{pipe}\;\mathrm{wall});\;$$

The shear stresses and pressure are assumed to be zero at the outlet.

Here, $V_{a}=Q/\left(\pi R_{1}^{2}\right)$ is a mean velocity over the channel cross-section, and Q is the flow rate (m^3^/s).

Boundary condition (5) is an ideal model with $K=\omega R_{1}/V_{a}$. In this study, we simplified this condition as follows:

(7) $$V_{\varphi }=\omega \cdot r\cdot \left(1-{\left(\frac{r}{R_{1}}\right)}^{30}\right),\;\omega =\frac{K\cdot V_{a}}{R_{1}}-\mathrm{angular}\;\mathrm{velocity}.\;$$

In this study, we use the two-mode Giesekus model [7].

(8) $$\sigma_{m}+\lambda_{m}\overset{\nabla }{\sigma_{m}}+\frac{\alpha_{m}\lambda_{m}}{\eta_{m}}\sigma_{m}\cdot \sigma_{m}=2\eta_{m}D,\left(m=1,\ldots ,2\right)$$

For the case of stationary flows $\frac{\partial \sigma }{\partial t}=0$, then the upper convective derivative takes the form $\overset{\nabla }{\sigma_{m}}=\left(\nabla \sigma_{m}\cdot V\right)-\left(\sigma_{m}\cdot \nabla V^{T}\right)-\left(\nabla V\cdot \sigma_{m}\right)$, $D=0.5\left(\Delta V+\Delta V^{T}\right)$ is the strain rate tensor; ($\lambda_{m}$,$\eta_{m}$) are the relaxation spectra, $\alpha_{m}$ is the rheological parameter of the Giesekus model. 

Equation (8), written in a cylindrical coordinate system for the considered case (axisymmetric formulation), is presented in the Appendix A.

As a specific liquid, we are going to consider DSM Stamylan LD 2008 XC43 low-density polyethylene (LDPE) melt with $\rho_{f}$ = 921 [kg/m^3^] [6]. The authors of [5] defined the parameters of the four mode Giesekus model, but such a high number of modes may cause a convergence problem. So, we defined the parameters of the two-mode Giesekus model (Table 1) for this fluid using the following algorithm. The pairs of $\left(\lambda_{i},\;\eta_{{}_{V_{i}}}\right)$ and $\eta_{{}_{N}}$ were found from:

(9) $$\begin{array}{l} F_{G}\left(\eta_{N},\eta_{V1},\ldots ,\eta_{Vm},\lambda_{1},\ldots ,\lambda_{m}\right)=\sum_{j=1}^{n}\left({\left[\left({G^{\prime }}_{j}-\sum_{i=1}^{m}\frac{\eta_{{}_{V_{i}}}\lambda_{i}{\left(\omega_{j}\right)}^{2}}{1+{\left(\lambda_{i}\right)}^{2}{\left(\omega_{j}\right)}^{2}}\right)/{G^{\prime }}_{j}\right]}^{2}\right.\\ \left.+{\left[\left({G^{\prime \prime }}_{j}-\eta_{{}_{N}}\omega_{j}-\sum_{i=1}^{m}\frac{\eta_{{}_{V_{i}}}\omega_{j}}{1+{\left(\lambda_{i}\right)}^{2}{\left(\omega_{j}\right)}^{2}}\right)/{G^{\prime \prime }}_{j}\right]}^{2}\right)\Rightarrow \min \end{array}$$

where $\left({G^{\prime }}_{j};\omega_{j}\right)$, $\left({G^{\prime \prime }}_{j};\omega_{j}\right)$ are experimental data, $\sum_{i=1}^{m}\frac{\eta_{{}_{V_{i}}}\lambda_{i}{\left(\omega_{j}\right)}^{2}}{1+{\left(\lambda_{i}\right)}^{2}{\left(\omega_{j}\right)}^{2}}$, $\eta_{{}_{N}}\omega_{j}+\sum_{i=1}^{m}\frac{\eta_{{}_{V_{i}}}\omega_{j}}{1+{\left(\lambda_{i}\right)}^{2}{\left(\omega_{j}\right)}^{2}}$ are numerical data, $\eta_{{}_{N}}$, $\eta_{{}_{V_{i}}}$, $\lambda_{i}$ are the solution to the optimization problem (9), *j* = 1…*n* is the number of an experimental point, and *m* is the mode number. 

We used the relative deviations of the experimental and calculated values of the dynamic moduli that allowed the improvement of the accuracy of the approximation [41]. The parameters $\alpha_{k}$ (*k* = 1, *m*) were calculated from:

(10) $$F\left(\alpha_{1},\ldots ,\alpha_{m}\right)=\sum_{j=1}^{n}{\left[\left(\tau_{j(}{}_{\mathrm{experiment})}-\tau_{j(}{}_{\mathrm{calc})}\right)/\tau_{j(}{}_{\mathrm{experiment})}\right]}^{2}\to \min,$$

where $\tau_{j(}{}_{\mathrm{calc})}=\eta_{N}{\left(\dot{\gamma }\right)}_{j}+\sum_{k=1}^{m}{\left(\tau_{k}\right)}_{j},\forall j=1,\ldots ,n$. j=1...n is the number of an experimental point, and k=1...m is the mode number.

The relation between the shear stresses of the *k*-th mode and the shear rate for a torsional flow of Giesekus fluid between two parallel plates (measurement system of rheometer) can be written as follows:

(11) $$2b_{k}{}^{2}\lambda_{k}{\left(\dot{\gamma }\right)}_{j}-2b_{k}\lambda_{k}{\left(\dot{\gamma }\right)}_{j}\sqrt{b_{k}{}^{2}-4\alpha_{k}{}^{2}{\left(\tau_{k}\right)}_{j}{}^{2}}+\;\left(\alpha_{k}\sqrt{b_{k}{}^{2}-4\alpha_{k}{}^{2}{\left(\tau_{k}\right)}_{j}{}^{2}+8\alpha_{k}b_{k}\lambda_{k}{\left(\dot{\gamma }\right)}_{j}{\left(\tau_{k}\right)}_{j}}+\alpha_{k}\sqrt{b_{k}{}^{2}-4\alpha_{k}{}^{2}{\left(\tau_{k}\right)}_{j}{}^{2}}\right){\left(\tau_{k}\right)}_{j}-2\alpha_{k}\lambda_{k}{\left(\dot{\gamma }\right)}_{j}b_{k}{}^{2}=0,\;(\forall k=1,\ldots ,m\;\mathrm{and}\;j=1,\ldots ,n),$$

where $b_{i}=\frac{\eta_{i}}{\lambda_{i}}$, $\dot{\gamma }$ is the shear rate (1/s), and τ is the shear stress (Pa).

**Figure 2:**
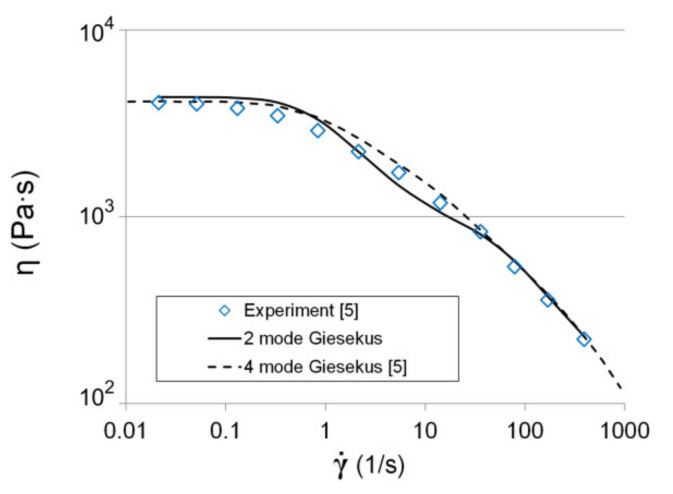
Viscosity curve: experiment and fitting with two and four-mode Giesekus model.

Figure 2 presents the comparison between experimental results and the numerical prediction of the viscosity curve. According to the well-known expression $\bar{\lambda }=\left(\sum_{k=1}^{2}\lambda_{k}^{}\eta_{k}\right)\cdot {\left(\sum_{k=1}^{2}\eta_{k}\right)}^{-1}$ and the obtained parameters of the two-mode Giesekus model, the relaxation time is equal to $\bar{\lambda }=0.84$ [s].

## 3. Approbation

**Figure 3:**
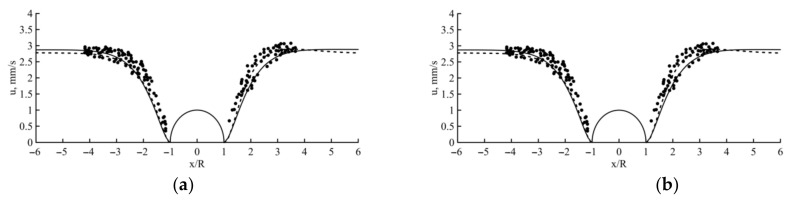
Cross-section axial velocities (**a**) and centerline velocity (**b**) solid line—4-mode Giesekus model with $\bar{\lambda }=1.74$, dashed line—2-mode Giesekus model with $\bar{\lambda }=0.84$, dots—experiment [5] (*V_a_* = 1.975 mm/s).

The obtained parameters of the two-mode Giesekus model were tested on a classic problem of a viscoelastic fluid flowing around a cylinder located between two infinite plates [5]. Numerical results were obtained using the OpenFoam with the viscoelastic package (planar flow). As Figure 3 shows, the distribution and value of the velocity components in the flow according to the two-mode Giesekus model are consistent with both experimental data and numerical results obtained using the four-mode Giesekus model.

The numerical implementation of the problem (1)–(8) is carried out in the Comsol Multiphysics package, which allows us to solve the custom equations by using the partial differential equations (PDE) package. The computational domain of the channel was subdivided using quadrangular elements with a minimum element quality of 0.78; the total number of elements was 45,300. The PARDISO method was used as a solver. The problem was solved on the XeonGold computational server with 24 cores and 512 Gb RAM.

**Figure 4:**
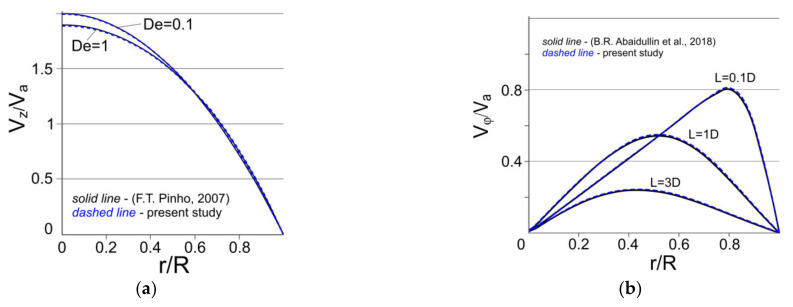
Comparison with literature data: (**a**) steady viscoelastic flow in a pipe [42], (**b**) swirl decay in a pipe (water, K = 1, Re = 98.8) [43].

As an approbation of our mathematical model (1)–(8), a comparison was made with the results of the problem-solution of the viscoelastic fluid flow in a circular channel (Figure 4a), and we also compared our results with the problem-solution of the circumferential velocity decay along the pipe length in a power-law fluid flow (Figure 4b). The figure shows good agreement between the calculated and published data; the approximation error does not exceed 0.9%. The test problems we have chosen have axial symmetry and are invariant with respect to the angle φ. In the literature, comparisons with the results of the problem of the viscoelastic fluid flow in a flat channel are given quite often, but the mathematical model we have developed does not allow us to study planar flows.

## 4. Results and Discussion

In this work, we considered the fixed geometry of the confusor: $L_{1}=1/4\cdot R_{1}$, $L_{2}=D_{1}={2R}_{1}$, $L_{3}=10D_{2}$, $D_{1}:D_{2}=4$ (contraction rate), $D_{1}=0.04$ m, $D_{2}=0.01$ m (Figure 1). According to the preliminary data, the length of the outlet part is $L_{3}=10D_{2}$ and it is sufficient for the outlet boundary conditions to have no effect on the solution inside the confusor.

**Figure 5:**
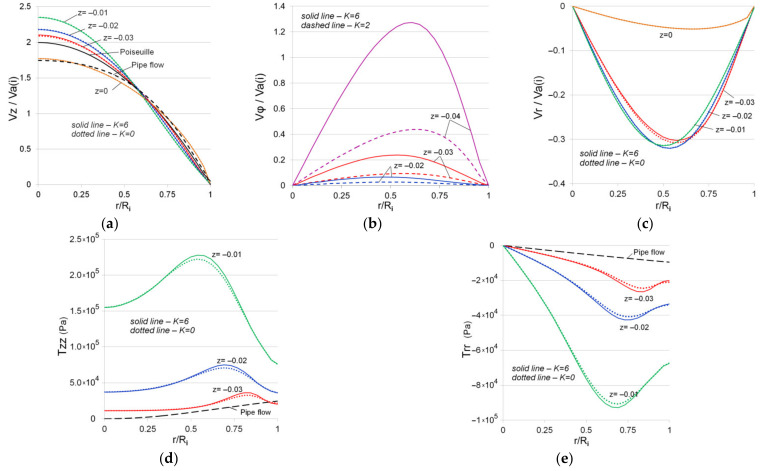
Normalized axial (**a**), circumferential (**b**) and radial (**c**) velocities distribution, normal stresses of extra stress tensor T (**d**,**e**) in the confusor for various initial swirl intensities, *Wi* = 5.04 (inlet) ($V_{a}$ = 0.03 m/s at the inlet).

Figure 5 shows the profiles of the normalized velocities: axial (a), circumferential (b), and radial velocity (c). The profiles shown in the figure are plotted in cross-sections of the channel (Figure 1), equidistant from each other at a distance of 0.01 m. It should be mentioned that the *z*-axis of the cylindrical coordinate system is centered in the cross-section corresponding to the outlet of the confusor and oriented in the flow direction. The components of the velocity vector are normalized by the mean velocity calculated for each cross-section $V_{a\;(i)}$ (*i* indicates the cross-section of the confusor). It is known that at a constant liquid flow rate, a decrease in the channel cross-section leads to an increase in mean velocity. For example, if for z = −0.045 (inlet) the velocity is given as $V_{a\;(inlet)}=0.03\;(m/s)$, then at z = −0.02 ($R_{1}\approx 0.01248\;(m)$) the mean velocity will increase to $V_{a\;(z=-0.02)}\approx 0.07704\;(m/s)$. All primary data were processed in Excel to reduce the accumulation of errors.

Figure 5 shows a comparison of the obtained results (K = 6) with similar results in a confusor without swirling (K = 0). As can be seen from the figure, the profile of the normalized axial velocity (Figure 5a) stretches as the fluid flows in the confusor due to the channel narrowing, then drops sharply and tends to the distribution corresponding to the steady flow in a round pipe. The behavior of axial velocity is similar to that in a channel with sudden contraction. An analysis of the obtained data revealed that the swirl intensity (parameter K in Equation (7)) affects only the circumferential velocity ($V_{\varphi }$), the distribution of which sharply tends to zero as the fluid flows in the confusor, i.e., the swirl intensity drops abruptly. The presence of radial velocity is a characteristic feature of fluid flow in a convergent channel (confusor) (Figure 5c). The figure demonstrates that the radial velocity is comparable to the axial one up to the value of z = −0.01 (3/4 of the confusor length).

Normal stress profiles for viscoelastic fluid flow in a convergent channel differ significantly from the similar profiles for fluid flow in a straight pipe (Figure 5c,d). For example, as the fluid flows in the confusor, the component $T_{zz}$ (a component of the extra stress tensor) takes on progressively higher values, with the maximum located in the middle region between the axis of symmetry and the channel wall. The $T_{rr}$ component over the channel cross-section, on the contrary, takes negative values, the highest absolute value of which is also concentrated in the region between the axis of symmetry and the channel wall. The presence of flow swirling (K = 6) insignificantly increases the value of the local extremum, which is consistent with the distribution of the velocity components.

**Figure 6:**
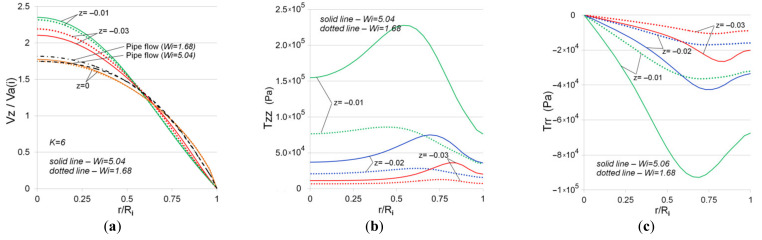
Normalized axial velocity (**a**) and normal stresses of extra stress tensor T (**b**,**c**) in the confusor for various initial swirl intensities, *Wi* = 5.04 (inlet) ($V_{a}$ = 0.03 m/s at the inlet).

The following figure illustrates the influence of the Weissenberg number on the distribution of axial velocity and normal stresses when the swirling viscoelastic fluid flows in a confusor. We considered *Wi* = 1.68 and *Wi* = 5.04 that were calculated by the formula $Wi=\bar{\lambda }V_{a}{\left(R_{2}\right)}^{-1}$ for the velocities $V_{a\;(\mathrm{inlet})}$ = 0.01 m/s and $V_{a\;(\mathrm{inlet})}$ = 0.03 m/s, respectively. Here, the value $V_{a\;(\mathrm{inlet})}$ is calculated for $R_{1\;(\mathrm{inlet})}$ = 0.02 (m). In the considered cross-sections, the greatest difference in the profiles of the normalized axial velocity is observed at z = −0.03, i.e., in the inlet region, which is apparently related to the selected boundary conditions at the channel inlet (Figure 6a). It was found (Figure 6b,c) that with an increase in the Weissenberg numbers, there is a significant increase in normal stresses, especially in the outlet region of the confusor. In this case, the stress profile with an increase in the Weissenberg number is characterized by the presence of a pronounced local extremum: for $T_{zz}$ this is a local maximum, for $T_{rr}$ this is a local minimum. In particular, for z = −0.01 the ratio $T_{zz\;(\max)}/T_{zz\;(\mathrm{axis}\;\mathrm{symmetry})}$ = 1.47 for *Wi* = 5.04 and ${\left|T_{rr\;}\right|}_{\left(\max\right)}/{\left|T_{rr\;}\right|}_{\left(\mathrm{axis}\;\mathrm{symmetry}\right)}$ = 1.12 for *Wi* = 1.68.

**Figure 7:**
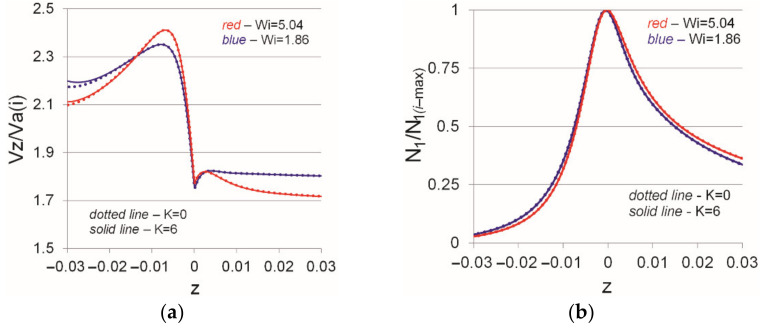
Normalized axial velocity (**a**) and first normal stress difference (**b**) along the centerline for various swirl intensities (K) and Weissenberg numbers (Wi).

Figure 7 most clearly illustrates the influence of the swirl intensity and the Weissenberg number on the flow structure in a convergent channel. The distributions of the normalized axial velocity and the first normal stress difference are plotted on the channel symmetry axis (central axis). It should be noted that, here, the current values of the axial velocity were also normalized by the actual mean velocity corresponding to a considered cross-section (meaning that as the fluid flows in the confusor, the mean integral flow velocity for the entire section increases due to the channel narrowing). As can be seen from Figure 7a, the flow of viscoelastic fluid is characterized by an increase in the axial velocity on the channel axis as it flows in the confusor, then a sharp drop and stabilization of the flow, i.e., after the outflow from the confusor, the velocity distribution tends to the one in steady pipe flow. The obtained results are in good agreement with similar results in the channel with sudden contraction. As can be seen from the figure, an increase in the Weissenberg number leads to a sharper increase in the axial velocity inside the confusor and the presence of a local maximum in the output region. As shown by the numerical results, for the considered case, the presence of flow swirl does not affect the distributions of both the axial velocity and the first normal stress difference. Note that $N_{1\;(i-\max)}$ was calculated for each distribution individually, so the maximum value of $N_{1\;}/N_{1\;(i-\max)}$ does not exceed unity. Similar to the axial velocity distribution, an increase in the Weissenberg number leads to a sharper increase in the value of $N_{1\;}/N_{1\;(i-\max)}$.

When studying swirling flows, it is convenient to use Equation (12) to estimate the swirl intensity [43].

(12) $$m(z)=\int_{0}^{1}v_{\varphi }v_{z}{\tilde{r}}^{2}d\tilde{r}/\int_{0}^{1}{\left(v_{z}\right)}^{2}\tilde{r}d\tilde{r}$$

where $v_{\varphi }=V_{\varphi }/V_{a}$, $v_{z}=V_{z}/V_{a}$, $\tilde{r}=r/R_{i}$ ($R_{i}$—for different cross-sections of the confusor). It should be noted that when substituting $v_{\varphi }=V_{\varphi }/V_{a}$ and $v_{z}=V_{z}/V_{a}$ in Equation (9), the value $V_{a}$ cancels out, so there is no need to recalculate $V_{a}$ for the cross-sections inside the confusor.

**Figure 8:**
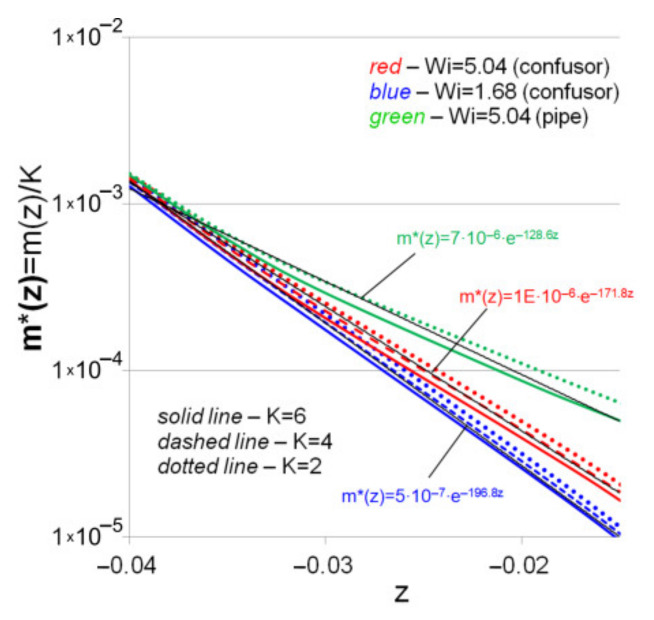
Axial variation of damping coefficients of swirling.

For the convenience of analysis, Figure 8 shows the distribution of m(z)/К, which made it possible to bring together the investigated relationships for K = 2, 4, 6. It can be seen from the figure that the dependences m*(z)=m(z)/К for *Wi* = 5.04 and *Wi* = 1.68 are satisfactorily described by the formula of the form $m*(z)=Ae^{B}$, which is consistent with the results of [43], in which the damping of the power-law fluid flow in a circular tube was studied. The calculations have shown that the presence of a confusor leads to a faster decrease in the swirl intensity. For example, in Figure 8, the results for the similar fluid flow in a straight pipe with the same boundary conditions at the inlet (*Wi* = 5.04) are shown in green. Thus, the presence of a convergent channel leads to the suppression of the swirl intensity and the flattening of the velocity profile.

## 5. Conclusions

In this paper, we developed a two-dimensional mathematical model of a steady laminar viscoelastic flow in the confusor under the condition of swirled flow imposed at the inlet. The parameters of the two-mode Giesekus model were obtained using the proposed relation. Approbation of the mathematical model showed good agreement with the literature data. It was observed that swirl intensity imposed at the inlet of the confusor with a contraction rate of 4:1 does not affect axial and radial velocities. The distribution of axial velocity along the axial direction is the following: it stretches along the channel axis and then becomes more flattened in the outlet section of the confusor. The magnitude of radial velocity is comparable to the magnitude of axial velocity up to 3/4 of the confusor length. The circumferential velocity distribution depends on boundary conditions; it decreases extensively along the axial direction and nearly disappears after 1/2 of the confusor length. A significant increase in normal stresses is observed with an increase in the Weissenberg number. Near the outlet of the confusor, the magnitudes of the T_zz_ component in the central region prevail over the corresponding magnitudes in the near-boundary region, which is fundamentally different from the flow of viscoelastic fluid in a straight pipe. The post processing of numerical results showed that the damping of the swirled flow in the confusor is more intense compared to a straight pipe. In this case, the distribution of damping coefficients of swirling along the length of the confusor can be described by an exponential function. The proposed mathematical model makes the estimation of hydrodynamic parameters a lot easier compared with a 3D statement.

## Figures and Tables

**Table 1:** Parameters of Giesekus model.

m	$\eta_{k}$ [Pa⋅s]	$\lambda_{k}\;[s]$	$\alpha_{k}$	$\eta_{N}$ [Pa⋅s]
1	694.01	0.01	0.495	88.05
2	3590.41	1	0.25	-

## Data Availability

Not applicable.

## Appendix A

Two mode Giesekus model for a particular case (axisymmetric swirl flow in the confusor)

$$\sigma_{rr(m)}+\lambda_{k}\left(V_{r}\frac{\partial \sigma_{rr(m)}}{\partial r}+V_{z}\frac{\partial \sigma_{rr(m)}}{\partial z}-2\sigma_{rr(m)}\frac{\partial V_{r}}{\partial r}-2\sigma_{rz(k)}\frac{\partial V_{r}}{\partial z}\right)+\frac{\alpha_{k}\lambda_{k}}{\mu_{k}}\left({\left(\sigma_{rr(m)}\right)}^{2}+{\left(\sigma_{r\varphi (m)}\right)}^{2}+{\left(\sigma_{rz(m)}\right)}^{2}\right)=2\mu_{k}\frac{\partial V_{r}}{\partial r},\;\sigma_{r\varphi (m)}+\lambda_{k}\left(V_{r}\left(\frac{\partial \sigma_{r\varphi (m)}}{\partial r}-\frac{\sigma_{r\varphi (m)}}{r}\right)+V_{z}\frac{\partial \sigma_{r\varphi (m)}}{\partial z}-\sigma_{r\varphi (m)}\frac{\partial V_{r}}{\partial r}-\sigma_{\varphi z(m)}\frac{\partial V_{r}}{\partial z}-\sigma_{rr(m)}\left(\frac{\partial V_{\varphi }}{\partial r}-\frac{V_{\varphi }}{r}\right)-\sigma_{rz(m)}\frac{\partial V_{\varphi }}{\partial z}\right)\;+\frac{\alpha_{k}\lambda_{k}}{\mu_{k}}\left(\sigma_{rr(m)}\sigma_{r\varphi (m)}+\sigma_{r\varphi (m)}\sigma_{\varphi \varphi (m)}+\sigma_{rz(m)}\sigma_{\varphi z(m)}\right)=\mu_{k}\left(\frac{\partial V_{\varphi }}{\partial r}-\frac{V_{\varphi }}{r}\right),\;\sigma_{rz(m)}+\lambda_{k}\left(V_{r}\frac{\partial \sigma_{rz(m)}}{\partial r}+V_{z}\frac{\partial \sigma_{rz(m)}}{\partial z}+\sigma_{rz(m)}\frac{V_{r}}{r}-\sigma_{zz(m)}\frac{\partial V_{r}}{\partial z}-\sigma_{rr(m)}\frac{\partial V_{z}}{\partial r}\right)\;+\frac{\alpha_{k}\lambda_{k}}{\mu_{k}}\left(\sigma_{rr(m)}\sigma_{rz(m)}+\sigma_{r\varphi (m)}\sigma_{\varphi z(m)}+\sigma_{rz(m)}\sigma_{zz(m)}\right)=\mu_{k}\left(\frac{\partial V_{r}}{\partial z}+\frac{\partial V_{z}}{\partial r}\right),\;\sigma_{\varphi \varphi (m)}+\lambda_{k}\left(V_{r}\left(\frac{\partial \sigma_{\varphi \varphi (m)}}{\partial r}-\frac{2}{r}\sigma_{\varphi \varphi (m)}\right)+V_{z}\frac{\partial \sigma_{\varphi \varphi (m)}}{\partial z}-2\sigma_{r\varphi (m)}\left(\frac{\partial V_{\varphi }}{\partial r}-\frac{V_{\varphi }}{r}\right)-2\sigma_{\varphi z(m)}\frac{\partial V_{\varphi }}{\partial z}\right)\;+\frac{\alpha_{k}\lambda_{k}}{\mu_{k}}\left({\left(\sigma_{r\varphi (m)}\right)}^{2}+{\left(\sigma_{\varphi \varphi (m)}\right)}^{2}+{\left(\sigma_{\varphi z(m)}\right)}^{2}\right)=2\mu_{k}\frac{V_{r}}{r},\;\sigma_{\varphi z(m)}+\lambda_{k}\left(V_{r}\left(\frac{\partial \sigma_{\varphi z(m)}}{\partial r}-\frac{\sigma_{\varphi z(m)}}{r}\right)+V_{z}\frac{\partial \sigma_{\varphi z(m)}}{\partial z}-\sigma_{rz(m)}\left(\frac{\partial V_{\varphi }}{\partial r}-\frac{V_{\varphi }}{r}\right)-\sigma_{zz(m)}\frac{\partial V_{\varphi }}{\partial z}-\sigma_{r\varphi (m)}\frac{\partial V_{z}}{\partial r}-\sigma_{\varphi z(m)}\frac{\partial V_{z}}{\partial z}\right)\;+\frac{\alpha_{k}\lambda_{k}}{\mu_{k}}\left(\sigma_{\varphi r(m)}\sigma_{rz(m)}+\sigma_{\varphi \varphi (m)}\sigma_{\varphi z(m)}+\sigma_{\varphi z(m)}\sigma_{zz(m)}\right)=\mu_{k}\frac{\partial V_{\varphi }}{\partial z},\;\sigma_{zz(m)}+\lambda_{k}\left(V_{r}\frac{\partial \sigma_{zz(m)}}{\partial r}+V^{\left\{z\right\}}\frac{\partial \sigma_{zz(m)}}{\partial z}-2\sigma_{k}{}^{\left\{rz\right\}}\frac{\partial V_{z}}{\partial r}-2\sigma_{zz(m)}\frac{\partial V_{z}}{\partial z}\right)\;+\frac{\alpha_{k}\lambda_{k}}{\mu_{k}}\left({\left(\sigma_{rz(m)}\right)}^{2}+{\left(\sigma_{\varphi z(m)}\right)}^{2}+{\left(\sigma_{zz(m)}\right)}^{2}\right)=2\mu_{k}\frac{\partial V_{z}}{\partial z}.$$
